# Implications of the Fast-Evolving Scale-Up of Adult Voluntary Medical Male Circumcision for Quality of Services in South Africa

**DOI:** 10.1371/journal.pone.0080577

**Published:** 2014-05-06

**Authors:** Dino Rech, Alexandra Spyrelis, Sasha Frade, Linnea Perry, Margaret Farrell, Rebecca Fertziger, Carlos Toledo, Delivette Castor, Emmanuel Njeuhmeli, Dayanund Loykissoonlal, Jane T. Bertrand

**Affiliations:** 1 Centre for HIV/AIDS Prevention Studies (CHAPS), Johannesburg, South Africa; 2 Tulane School of Public Health and Tropical Medicine, New Orleans, Louisiana, United States of America; 3 United States Agency for International Development, Pretoria, South Africa; 4 Centers for Disease Control and Prevention, Pretoria, South Africa; 5 United States Agency for International Development, Washington, DC, United States of America; 6 South African National Department of Health, South Africa; World Health Organization, Switzerland

## Abstract

**Background:**

The scale-up of voluntary medical male circumcision (VMMC) services in South Africa has been rapid, in an attempt to achieve the national government target of 4.3 million adult male circumcisions for HIV prevention by 2016. This study assesses the effect of the scale-up on the quality of the VMMC program.

**Methods and Findings:**

This analysis compares the quality of services at 15 sites operational in 2011 to (1) the same 15 sites in 2012 and (2) to a set of 40 sites representing the expanded program in 2012. Trained clinicians scored each site on 29 items measuring readiness to provide quality services (abbreviated version of the WHO Quality Assessment [QA] Guide) and 29 items to assess quality of surgical care provided (pre-op, surgical technique and post-op) based on the observation of VMMC procedures at each site. Declines in quality far outnumbered improvements. The negative effects in terms of readiness to provide quality services were most evident in expanded sites, whereas the declines in provision of quality services tended to affect both repeat sites and expanded sites equally. Areas of notable concern included the monitoring of adverse events, external supervision, post-operative counselling, and some infection control issues. Scores on quality of surgical technique tended to be among the highest across the 58 items observed, and the South Africa program has clearly institutionalized three “best practices” for surgical efficiency.

**Conclusions:**

These findings demonstrate the challenges of rapidly developing large numbers of new VMMC sites with the necessary equipment, supplies, and protocols. The scale-up in South Africa has diluted human resources, with negative effects for both the original sites and the expanded program.

## Introduction

South Africa has the highest number of HIV-infected people in the world (5.6 million people) and an HIV prevalence of 17.3% [1]. After randomized clinical trials demonstrated voluntary medical male circumcision (VMMC) to be effective in reducing HIV transmission [2]–[4], South Africa was among the 14 countries prioritised for a rapid scale-up of VMMC services [5] because of the low male circumcision prevalence (approximately 42%) [6] and the high HIV prevalence [7]. Mathematical modelling data showed that achieving 80% VMMC coverage in South Africa requires the circumcision of 4.3 million men aged 15 to 49 years by 2016 [8]. For every five VMMC procedures performed in South Africa, one new HIV infection is averted [9]. Recent research in the Orange Farm community (the site of the South African clinical trial) supports the hypothesis from mathematical modelling; HIV prevalence was observed to decline from 12.5% to 9.3% among males aged 15 to 49 years, and the corrected HIV incidence rate was 2.86/100 person-year among uncircumcised men and 0.42/100 among circumcised men in the 15 to 35 year age group [10].

In an effort to streamline the VMMC procedure and maximize efficiencies, a consultation of experts convened by the World Health Organization (WHO), developed a document known as the Models for Optimizing the Volume and Efficiency for Male Circumcision Services (or MC MOVE) [11]. The WHO has also outlined the program quality required for scale-up of VMMC services in the WHO VMMC Quality Assurance (QA) Guide [12], which focuses on the quality of services, both in terms of the actual operative procedure and the provision of the minimum package of services associated with a VMMC procedure. This minimum package includes the provision of HIV and VMMC education, HIV testing and counselling, medical screening prior to surgery, safe surgical procedure, post-operative instructions and recovery, follow-up and emergency services, as well as safe and appropriate supplies and medications throughout the steps above in a clean and suitable environment. With multiple components, VMMC is complex, making the rapid scale-up of services challenging. Further, the rapid scale-up of any intervention is often associated with various challenges, which impact on the quality of services offered [13]. Ideally, the provision of any scaled-up and high-volume efficient service should not lower the quality or safety of the procedure [14].

SYMMACS (the Systematic Monitoring of the Voluntary Medical Male Circumcision Scale-up in Eastern and Southern Africa) systematically assessed the quality and safety of the VMMC scale-up in four countries: South Africa, Kenya, Tanzania, and Zimbabwe. Of the four, South Africa has had the most rapid scale-up (from one VMMC site in 2010 to over 80 sites by 2012). Moreover, it has performed the largest number of procedures (approximately 30% of all VMMCs in the 14 priority countries between 2008 and 2011) [15]. The objective of this analysis is to determine the effects of the rapid expansion in South Africa on two aspects of quality: (1) readiness of VMMC facilities to provide quality services and (2) actual quality of surgical care provided. The null hypothesis is that program expansion did not affect service quality.

## Methods

The SYMMACS study design and methods are described in detail elsewhere [16]. In 2011, all VMMC sites known to be operational (at which data collection was feasible) were selected, resulting in 15 sites. In 2012 the sample included the 15 original (“repeat”) sites, plus 25 new sites that were purposefully sampled from over 80 sites in operation. Criteria for selection of new sites included (1) geographic diversity (six of the nine provinces in South Africa) and (2) high volume facilities, defined as performing at least 100 procedures per month or 10 procedures per day of operation. This process yielded 40 VMMC sites in 2012, labelled “expanded sites”. The current analysis draws on data from three of the four SYMMACS instruments: an abbreviated version of the WHO Quality Assessment of facilities [17] (Table 1), observation of VMMC procedures (Table 2, 3, and 4), and interviews with providers (Table 5).

**Table 1:** Quality assessment of VMMC sites (measuring readiness to provide quality services), by year.

Item observed	2011: n = 15 sites									p-value	
	2012a: n = 15 sites (same site comparison)									(Fisher's exact test)	
	2012b: n = 40 sites (expanded site comparison)										
	0 = Unsatisfactory			1 = Partially			2 = Satisfactory				
	%			satisfactory %			%				
VMMC sites	2011	2012a	2012b	2011	2012a	2012b	2011	2012a	2012b	2011 v. 2012a	2011 v. 2012b
**Characteristics**											
Light in surgical area	0.0	0.0	0.0	6.7	0.0	7.5	93.3	100	92.5	1.000	1.000
Ventilation in surgical area	6.7	0.0	2.5	6.7	13.3	25.0	86.7	86.7	72.5	1.000	0.202
General appearance of VMMC facility	0.0	0.0	0.0	13.3	26.7	45.0	86.7	73.3	55.0	0.651	0.057
**Adequacy of information systems**											
Existence of functioning information system	0.0	0.0	0.0	40.0	0.0	2.5	60.0	100	97.5	0.017	0.001
VMMC client consent forms on file	6.7	0.0	2.5	0.0	0.0	0.0	93.3	100	97.5	1.000	0.475
Monitoring system in place for adverse events	26.7	40.0	50.0	13.3	33.3	27.5	60.0	26.7	22.5	0.209	0.046
**Education, counseling and referral**											
Group education on risks & benefits of VMMC	6.7	6.7	12.5	0.0	0.0	7.5	93.3	93.3	80.0	1.000	0.571
Individual HTC & question time on VMMC	0.0	7.7	10.0	0.0	0.0	22.5	100	92.3	67.5	0.464	0.022
Referral slips for clients	13.3	40.0	47.5	0.0	13.3	10.0	86.7	46.7	42.5	0.063	0.013
**Supervisory mechanisms**											
Report of supervisory visits in past 6 months	26.7	60.0	72.5*	13.3	0.0	10.0	60.0	40.0	17.5*	0.0841	0.004
External monitoring of AEs in past 6 months	60.0	93.3	92.5	0.0	0.0	5.0	40.0	6.7	2.5	0.0801	0.002
**Availability of protocols**											
WHO guidelines for performing VMMC	60.0	33.3	45.0	0.0	13.3	12.5	40.0	53.3	42.5	0.247	0.442
National STI protocols	26.7	53.3	62.5	0.0	0.0	7.5	73.3	46.7	30.0	0.264	0.016
**Availability of operating supplies & equipment**											
Sterilized VMMC instruments	13.3	0.0	2.5	0.0	0.0	2.5	86.7	100	95.0	0.483	0.406
Correctly stored & unexpired local anaesthesia	0.0	0.0	0.0	0.0	6.7	10.0	100	93.3	90.0	1.000	0.566
Antibiotics for VMMC/AEs in stock	13.3	0.0	5.1	6.7	28.6	35.9	80.0	73.3	59.0	0.182	0.048
Pain medication in stock	0.0	0.0	2.5	0.0	13.3	22.5	100	86.7	75.0	0.483	0.061
Antiseptics in stock	0.0	0.0	0.0	0.0	6.7	12.5	100	93.3	87.5	1.000	0.308
Dressing materials (bandages & gauze)	0.0	0.0	5.1	0.0	14.3	15.4	100	85.7	79.5	0.224	0.230
Sharps container in surgical area	0.0	0.0	2.5	0.0	0.0	2.5	100	100	95.0	1.000	1.000
**Availability of CPR equipment**											
CPR bag mask	26.7	40.0	50.0	6.7	6.7	2.5	66.7	53.3	47.5	0.699	0.140
Oxygen supply	26.7	33.3	45.0	0.0	6.7	7.5	73.3	60.0	47.5	0.548	0.128
IV lines & fluids	26.7	6.7	22.5	0.0	0.0	0.0	73.3	93.3	77.5	0.329	1.000
Antihistamine	13.3	13.3	17.5	0.0	26.7	27.5	86.7	60.0	55.0	0.091	0.024
**Availability of prophylactic supplies**											
Post-exposure infection prophylaxis	40.0	46.7	40.0	0.0	40.0	52.5	60.0	13.3	7.5	0.003	<0.0001
Guidelines for post-exposure prophylaxis	13.3	64.3	67.5	0.0	0.0	0.0	86.7	33.3	32.5	0.008	<0.0001
**Availability of HTC protocols &supplies**											
Male condoms availability	13.3	33.3	27.5	0.0	0.0	2.5	86.7	66.7	70.0	0.389	0.499
HTC provision	0.0	0.0	20.0	0.0	13.3	12.5	100	86.7	67.5	0.483	0.049
HTC audio/visual privacy	6.7	6.7	22.5	0.0	6.7	17.5	93.3	86.7	60.0	1.000	0.067
**Summary of QA scores**	**n = 15 sites**			**n = 15 sites**			**n = 40 sites**			**p-value**	
% of QA items where all sites satisfactory	73.3			40			17.5			0.139	<0.0001
Mean QA score across all parameters	1.68			1.51			1.36			0.086	0.0003

* Eight sites were visited by external supervisory teams shortly after the SYMMACS data collection, which is not reflected in these data.

**Table 2:** Assessment of pre-operative and safety control procedures for VMMC, by year.

Item observed	2011: n = 116 procedures									p-value	
	2012a: n = 150 procedures (same site comparison)										
	2012b: n = 361 procedures (expanded site comparison)										
	0 = Unsatisfactory			1 = Partially			2 = Satisfactory				
	%			satisfactory %			%				
	2011	2012a	2012b	2011	2012a	2012b	2011	2012a	2012b	2011 v. 2012a	2011 v. 2012b
**Pre-operative assessment:**											
Clinical personnel conduct basic preoperative assessment including a targeted history & physical exam to exclude surgical contraindications, primarily bleeding disorders, allergies, immune-compromised states & STIs	33.6	50.0	53.8	14.3	29.7	34.0	51.2	20.3	12.3	<0.0001	<0.0001
**Surgical procedures sterility management**											
Sterile instruments & consumables used	0.8	0.0	0.6	0.8	0.0	0.3	98.3	100	99.1	0.197	0.365
Sterile gloves used	3.3	8.3	5.4	1.7	13.8	8.2	95.0	77.9	86.4	<0.0001	0.018
Hand washing/disinfection between clients	8.3	33.8	25.8	1.7	13.4	14.7	90.0	52.8	59.5	<0.0001	<0.0001
Maintenance of an adequate sterile surgical operating field	5.9	9.7	4.3	7.6	29.9	16.8	86.4	60.4	78.9	<0.0001	0.036
Use of protective eyewear	80.7	81.5	86.0	8.4	14.4	8.7	10.9	4.1	5.3	0.043	0.126
Safe, secure storage & disposal of medical waste by provider	2.5	2.0	4.5	5.0	23.0	22.9	92.4	75.0	72.6	<0.0001	<0.0001
Correct & hygienic instrument processing	2.5	0.7	0.6	0.0	4.2	1.7	97.5	95.1	97.7	0.019	0.080
Disinfection of surgical beds & area between clients	3.3	12.2	8.7	4.2	25.0	30.4	92.5	62.8	60.8	<0.0001	<0.0001

**Table 3:** Assessment of quality of surgical technique for VMMC, by year.

Item observed	2011: n = 116 procedures									p-value	
	2012a: n = 150 procedures (same site comparison)										
	2012b: n = 361 procedures (expanded site comparison)										
	0 = Unsatisfactory			1 = Partially			2 = Satisfactory				
	%			satisfactory %			%				
	2011	2012a	2012b	2011	2012a	2012b	2011	2012a	2012b	2011 v. 2012a	2011 v. 2012b
**Assessment of surgical procedures**											
Clean surgical area with recommend scrub solution	0.0	0.7	0.6	0.0	17.4	9.5	100	81.9	89.9	<0.0001	<0.0001
Correctly identify the skin to be excised	0.0	15.0	15.8	1.7	12.2	12.7	98.3	72.8	71.5	<0.0001	<0.0001
Demonstrate the “safety first approach” to ensure no part of penis besides the foreskin is in danger of being injured	0.0	0.0	0.0	2.5	2.7	2.8	97.5	97.3	97.2	1.000	1.000
Demonstrate safe administration of local anaesthesia	3.6	2.0	1.4	0.0	27.0	27.9	96.4	70.9	70.8	<0.0001	<0.0001
Demonstrate cautious & gentle approach to removing the foreskin	0.0	0.0	1.1	3.4	6.7	3.6	96.6	93.3	95.3	0.276	0.742
Adequately controls bleeding with electrocautery and/or ligating sutures	0.0	0.0	0.8	0.0	4.7	3.1	100	95.3	96.1	0.019	0.79
Use correct technique to tie surgical knots	21.2	9.4	9.5	4.2	20.8	28.2	74.6	69.8	62.3	<0.0001	<0.0001
Correctly align the frenulum and places secure mattress suture	0.0	1.3	1.4	0.0	2.7	10.3	100	96.0	88.3	0.085	<0.0001
Correctly align the other quadrant sutures	1.7	0.0	0.0	1.7	4.7	2.8	96.6	95.3	97.2	0.125	0.082
Avoid placing deep sutures around the frenulum	0.0	0.7	0.3	0.8	1.3	2.8	99.2	98.0	96.9	1.000	0.479
Place interrupted sutures evenly to avoid leaving gapping margins	5.2	1.3	1.1	5.2	3.4	1.9	89.6	95.3	96.9	0.149	0.005
Ensure no significant bleeding present	0.0	0.0	1.1	0.8	0.0	2.3	99.2	100	96.6	0.447	0.508
Place a secure dressing that's not excessively tight	0.0	0.0	2.0	0.0	0.0	0.8	100	100	97.2	1.000	0.267

**Table 4:** Assessment of post-operative procedures for VMMC, by year.

Item observed	2011: n = 116 procedures									p-value	
	2012a: n = 150 procedures (same site comparison)										
	2012b: n = 361 procedures (expanded site comparison)										
	0 = Unsatisfactory			1 = Partially			2 = Satisfactory				
	%			satisfactory %			%				
	2011	2012a	2012b	2011	2012a	2012b	2011	2012a	2012b	2011 v. 2012a	2011 v. 2012b
**Assessment of post-operative procedures for VMMC**											
Staff observe post-op clients for an allergic reaction/any other abnormality before allowing them to leave the operating table/recovery room	54.2	83.2	84.7	4.2	7.4	7.8	41.5	9.4	7.5	<0.0001	<0.0001
Staff review vital signs	32.2	39.2	39.4	0.0	2.7	1.4	67.8	58.1	59.2	0.097	0.159
Staff provide patients with clear instructions, verbal & written on how to wash & care for the wound and how to deal with pain & minor bleeding	7.6	14.8	17.0	12.6	41.6	37.6	79.8	43.6	45.4	<0.0001	<0.0001
Staff insist/encourage clients to return for a follow-up visit within 48 hours of the VMMC/in the case of a complication	10.1	28.4	34.4	1.7	2.0	2.0	88.2	69.6	63.7	<0.0001	<0.0001
Staff provide emergency contact details to clients	32.8	62.2	64.5	0.0	0.0	0.0	67.2	37.8	35.5	<0.0001	<0.0001
Patients receive post-operative counselling instructions & reinforcement of previous VMMC/HIV messaging	85.6	94.0	95.3	4.2	0.7	0.3	10.2	5.3	4.5	0.038	<0.0001
Staff give specific reminders of the 6 week post-operative abstinence period	47.1	71.1	70.7	0.0	1.3	0.8	52.9	27.5	28.5	<0.0001	<0.0001

**Table 5:** Provider characteristics, operating times, and efficiency elements across repeat and expanded sites.

	2011	2012	2012	p-value	
	15 sites	15 sites (same site comparison)	40 sites (expanded site comparison)		
**Provider characteristics** 1	**n = 105 providers**	**n = 85 providers**	**n = 209 providers**	**2011 v. 2012a**	**2011 v. 2012b**
% of providers that have completed additional training (eg. certificate training) in VMMC for HIV prevention	100	80	76.6	<0.0001	<0.0001
Mean number of months of experience performing VMMC for HIV prevention	19.2 months	15.9 months	12.7 months	0.465	0.139
In the past 3 months % providers that performed VMMC:					
–Full-time (at least 90% of working hours)	80.0	80.0	78.5		
–Part-time	20.0	20.0	21.5	1.000	0.883
**Efficiency elements** 1	**n = 105 providers**	**n = 85 providers**	**n = 209 providers**		
% providers who have ever used electrocautery/diathermy for haemostasis in performing/assisting in VMMC	99.0%	97.6%	98.1%	0.588	0.668
% providers that report using pre-bundled instruments & supplies in past 3 months	99.0%	100%	100%	1.000	0.334
Mean number of beds per site	4.8 beds	4.1 beds	3.7 beds	0.621	0.047
**Operating time (mean duration in minutes:seconds)** 2	**n = 120 procedures**	**n = 150 procedures**	**n = 361 procedures**		
Primary provider time with client (foreskin removal, haemostasis, primary provider sutures)	06∶19	06∶13	06∶45	0.818	0.418
Total operating time (scrubbing to cleaning)	23∶26	25∶52	25∶43	0.0006	0.0001

1 Based on responses from provider survey;

2 Based on observation of VMMC procedures.

A team of VMMC-trained clinicians made a two-day visit to each selected site in 2011 and 2012. The core data collection team remained the same across both years of the study, with the exception of one team member, who left the project at the end of 2011 and was replaced in 2012. The team assessed “readiness to offer quality services” based on the observation of 29 items measuring availability of equipment, supplies, and protocols, as well as infrastructure. The team evaluated “quality of surgical care provided” based on the observation of up to 10 VMMC procedures per site, including pre-op procedures, surgical technique, and post-op procedures (29 items). Each item was scored on a 3-point scale, where, ‘0’ was unsatisfactory, ‘1’ was partially satisfactory and ‘2’ was satisfactory, based on written criteria. The data collectors recorded scores individually and then compared and discussed their scores in order to reach a consensus on the final score provided for each item. Additional data were obtained on characteristics of the providers, operating time per procedure, and the extent of adoption of surgical efficiency elements (instruments are available online [18]). Data were entered manually, then transferred to personal digital assistants (PDAs) and analysed using SPSS version 19.0 (IBM Corporation).

The percentage distributions for each item (scored as 0, 1, or 2) were tested for differences at (1) the original (repeat) sites between 2011 and 2012, that would indicate improvement or decline in quality, and (2) between the 15 repeat sites and the 40 “expanded” sites, which would indicate improvement or decline in the program as a whole. Several non-parametric statistical tests were employed. Fisher's exact test was used to examine differences between years (2011 versus 2012) on items in the facility assessment (Table 1), observation of the VMMC procedures (Table 2), and four items from the provider interview (Table 4). The Kruskal-Wallis one-way ANOVA tested for differences by year in the time required to complete the operation (Table 4). Parametric t-tests were used to analyse data pertaining to providers' experience in VMMC and the mean number of beds at VMMC sites (Table 4). The analysis also tested for differences between 2011 and 2012 (repeat and expanded sites) on two summary measures: (1) an overall quality index score reporting the percentage of sites that received a satisfactory score (“2”) on at least 22 of the 29 items (thus 75%), using chi square, and (2) the mean quality score across all items (averaging the scores of ‘0’, ‘1’ and ‘2’ for each item), based on t-tests. Significant differences refer to p-values<0.05 unless otherwise specified.

Human subjects approval for this study was obtained from the Tulane University Institutional Review Board (IRB) and from the University of the Witwatersrand's Human Research Ethics Committee.

## Results

A total of 122 VMMC procedures were observed at the 15 original sites in 2011; 361 procedures were observed at the 40 sites in 2012. The results for specific items fall into the following categories: (1) no change; (2) improvement at repeat sites, expanded sites, or both; or (3) decline in quality at repeat sites, expanded sites, or both. Decreases in quality far outweighed any improvements (found on only 2 of 58 items observed).

### Quality assessment of the facilities (observation of the sites)

In terms of readiness to provide quality services (i.e., the extent to which sites have the necessary equipment, supplies, infrastructure, guidelines and other materials), in 2011 the original 15 sites scored “satisfactory” on almost three-quarters (73%) of the items observed, indicating a generally strong performance but with areas for improvement (see Table 1).

As of 2012, there was no change on 17 of the 29 items. On 9 of 29 items, quality declined at the expanded but not the repeat sites. Two additional items showed decline at both repeat and expanded sites, and only one item showed improvement. The nine items with a significant decrease in quality at expanded sites but not at repeat sites involved diverse aspects of quality: monitoring systems in place for adverse events (AEs) (either manual or computerized systems that record the types and severity of adverse events seen), individual HTC and question time on VMMC, referral slips given to clients for services not available onsite, availability of national STI protocols onsite, antibiotics for VMMC/adverse events in stock, antihistamine for cardiopulmonary resuscitation (CPR), and the provision of HIV counselling and testing. Two additional items showing decline at expanded sites included a report of a supervisory visit in the past six months and external monitoring of adverse events in the past six months; however, some sites had been in operation for less than six months. The two items with decreased quality at both repeat and expanded sites were availability of post-exposure prophylaxis and guidelines for post-exposure prophylaxis. Of the 17 items showing no change, it is important to flag items that were low in 2011 and remained low in 2012 at both repeat and expanded sites: availability of WHO guidelines onsite for performing VMMC, availability of CPR equipment (CPR bag mask and oxygen supply), and availability of male condoms. The only item showing improvement – at both repeat and expanded sites – was the existence of a functioning information system (manual or computerized).

The summary measures shown at the bottom of Table 1 capture the key findings related to readiness of VMMC sites to provide quality services: significant declines occurred between 2011 and 2012 in the sample of expanded sites. Specifically, in 2011 73% of the items were scored as satisfactory at the 15 sites; this dropped to 40% (repeat sites) and 18% (expanded sites) in 2012, though the difference was only statistically significant in expanded sites. Similar results were obtained on the mean QA score across the 29 items, which dropped from 1.68 (in 2011) to 1.51 (repeat sites; borderline significant) and to 1.36 (expanded sites; significant).

### Assessment of quality of services provided (observation of VMMC procedures)

Tables 2–4 present findings on the quality of services provided, based on the observation of pre-op procedures (9 items), surgical technique (13 items) and post-op procedures (7 items) performed by providers.

#### Pre-op and safety control procedures

As of 2011, the 15 original sites scored above 75% on seven of the nine items. On six of the nine items, quality declined significantly between years at both the repeat and expanded sites; items included clinical personnel conduct a basic preoperative assessment, sterile gloves used, hand washing/disinfection between clients, maintenance of an adequate sterile surgical field while operating, safe secure storage and disposal of medical waste by provider, and disinfection of surgical beds and areas between clients. In addition, quality declined significantly at repeat sites but not at expanded sites on two items: the use of protective eyewear and correct/hygienic instrument processing. No change was noted in the use of sterile instruments and consumables.

#### Quality of surgical technique

Table 3 presents the findings on 13 items used to assess the quality of the surgical technique during the VMMC procedure, measuring technical competence of providers and the care and safety with which surgical steps were undertaken. Of all the QA measures presented in this analysis, the scores for surgical technique were among the highest in this assessment, across both years and across repeat and expanded sites. Specifically, in 2011 at least 90% of providers scored “satisfactory” on 12 of the 13 items, the one exception being the use of the correct technique to tie surgical knots. Despite the generally high scores on surgical technique, significant changes occurred between 2011 and 2012. Quality actually improved at expanded sites on “placing interrupted sutures evenly to avoid leaving gaping margins”. Quality scores dropped at both repeat and expanded sites on four items: cleaning the surgical area with a recommended scrub solution, correctly identifying the skin to be excised, safe administration of local anaesthesia, and use of correct technique to tie surgical knots. Additional declines included correctly aligning the frenulum and placing secure mattress sutures (expanded but not repeat sites) and adequately controlling bleeding with electrocautery and/or ligating sutures (at repeat but not expanded sites). No significant changes occurred on the remaining four items.

#### Assessment of post-op procedures

Seven items were observed. In 2011, scores were already low on five of the seven scores (less than 75% of providers scoring satisfactory). Yet between 2011 and 2012 they declined further, at both repeat and expanded sites on six of the seven items: observing clients for allergic reaction/any other abnormality before they leave the table or recovery area, providing clear instructions on post-op care and pain, encouraging return for follow-up, providing emergency contact details, giving post-op counselling including reinforcement of previous messaging, and reminding clients of the six-week abstinence period. On the item with no change (review of vital signs), scores remained low in both years.

In sum, the main findings on quality of services provided (based on observation of pre-op procedures, surgical technique, and post-op procedures by providers) were significant declines at both repeat and expanded sites on 16 of 29 items, with one additional decline (each) for repeat sites and for expanded sites.

### Characteristics of VMMC providers, operating time, and adoption of surgical efficiency elements

The percentage of providers that reported to have received training in VMMC for HIV prevention decreased significantly between the two years, from 100% in 2011 to 80% (repeat sites) and 77% (expanded sites) in 2012 (Table 5). The months of experience of providers conducting (or assisting in) VMMC also decreased, from a mean of 19.2 months in 2011 to 15.9 months (repeat sites) and 12.7 months (expanded sites) in 2012, although this change was not statistically significant due to large variability within the data.

Table 5 also presents data on three of the six elements of surgical efficiency tested under SYMMACS: rotation among multiple surgical bays, the use of electrocautery to stop bleeding (instead of ligating sutures), and the use of pre-bundled kits with disposable instruments. These data reflect the almost universal adoption of electrocautery and use of pre-bundled kits with disposable instruments in the South African program in both years and at all sites. The mean number of surgical beds decreased significantly from 4.8 in 2011 to 3.7 at expanded sites in 2012; but even this number reflects widespread use of multiple surgical bays. In short, providers adhered to these three “best practices” in surgical efficiency.

The average (mean) time the primary provider spent with the client (on the crucial steps of removing the foreskin, stopping the bleeding with electrocautery, and applying the mattress sutures) was 6∶19 minutes in 2011, compared to 6∶13 minutes (repeat sites) and 6∶45 seconds (expanded sites) in 2012. There was no significant difference by year or between repeat/expanded sites; it averaged between 6–7 minutes. On the second measure, total elapsed operating time, the mean increased significantly from 23∶26 minutes at the 15 original sites (2011) to close to 26 minutes at both repeat and expanded sites in 2012.

## Discussion

The results of this study show the difficulties of maintaining quality with the rapid scale-up of VMMC programming in South Africa. The negative effects, in terms of readiness to provide quality services were most evident with regard to the expanded sites, whereas the declines in provision of quality services tended to affect both repeat sites and expanded sites equally.

The findings on readiness (e.g., the extent to which sites have the necessary equipment, supplies, infrastructure, guidelines and other materials) suggest that with the rapid scale-up in sites, it was simply not possible to establish the same level of readiness in new sites as had been available in the original sites. The only improvement – seen in both repeat and expanded sites – involved the existence of a functional information system, needed for accurately tracking the main indicator of performance in VMMC programs: number of VMMCs completed per month. The only area where repeat sites showed a decline in the readiness to provide quality services was the post-exposure infection prophylaxis and guidelines for its use, which many sites had in adjoining buildings but not in the actual operating theatre. Low scores on availability of emergency CPR equipment appear to result from provider perceptions that the risk of emergency situations is low and that help will be available from adjacent health clinics, even if emergency equipment and supplies are not available in the operating theatre. However, the occurrence of a life-threatening situation during SYMMACS data collection in 2012 underscored the critical importance of having emergency equipment and supplies close at hand.

The declines observed in provision of quality services at both repeat and expanded sites (on 16 of 29 items) reflect a dilution of resources in the process of scale-up. At the start of the program in 2011, resources (including provider expertise) tended to be concentrated in regional sites, such as Orange Farm; the site of the South African clinical trial and the first high volume VMMC site. However, as the program expanded in 2012 to many new sites, it was necessary to deploy these skilled personnel to train others and set up additional sites. This process drained resources from the original sites, without developing the same level of expertise in the new sites; supervision decreased, as shown herein. Data on provider characteristics further supports this explanation. Whereas 100% of providers in 2011 had received specialized VMMC training, this percentage decreased significantly to 80% (repeat sites) and 77% (expanded sites) in 2012, further indicating the negative effects of expansion at repeat sites.

The challenges to rapid scale-up are not unique to VMMC in South Africa. In relation to the scale-up of intervention packages for child survival across six sub-Saharan African countries, Oliver et al [13] reported similar challenges with supervision, supply chain systems, and weak monitoring and evaluation systems. Problems with monitoring and evaluation systems also occurred with the scale-up of an HIV care and support program in Chennai, India [19] and in a family planning program scale-up introducing injectable contraceptives in Vietnam [20].

As one of 14 countries prioritised for VMMC scale-up, South Africa is under great pressure from stakeholders (e.g., government and funders) to perform large numbers of circumcisions. Consequently, programs give priority to maintaining information systems that capture the number of VMMCs performed in contrast to information systems that record adverse events, which are thought to reflect negatively on programs. The SYMMACS data collected using existing systems intended to track adverse events in all four countries; in South Africa they were of such poor quality that they were excluded from the analysis. Given the importance of AEs as an indicator of safety, governments should continue to refine their systems for tracking and reporting AEs.

Of the four main segments of the QA (facility assessment, pre-op procedures, surgical technique and post-op procedures), the scores were highest for surgical technique, despite the observed limitations in supplies, equipment, and materials in the expanded sites. By late 2012, over 2,000 providers had completed the standardised South African VMMC training course, based on the WHO surgical guidelines. The near universal adoption in South Africa of three elements of surgical efficiency (rotation among surgical beds, use of pre-bundled instruments with disposable supplies, and use of electrocautery) is noteworthy. Despite shortcomings on readiness and quality of surgical care documented above, the South African program has managed to institutionalize these “best practices” throughout its program, including at the expanded sites. However, the South African government does not authorize task-shifting, which is considered to be a key determinant in operating theatre efficiency (see [21], [22] in this supplement).

These findings demonstrate that rapid program expansion, especially over a short time period, needs to be considered in conjunction with a plan to maintain quality. Program planners should consider supply chain and logistical issues and focus on emergency supplies, which are often neglected. A note needs to be made to ensure good monitoring systems are in place for all program aspects, including the monitoring of adverse events. External supervisory mechanisms need to be implemented, improved and expanded as the program grows. On the basis of these findings and results from other research, the government has convened a VMMC technical working group in monitoring and evaluation who are responsible for overseeing the roll-out of additional supervision and quality assurance measures in 2013, in an effort to address many of the issues highlighted in this paper.

### Limitations

It was not possible to know the “universe” of VMMC sites in South Africa. In 2011, all sites known to be operational and where authorization could be obtained were included. By 2012, an estimated 80 sites became operational, but there was no accurate or complete listing available. Thus, new sites were included using purposeful (rather than random) selection. Although written criteria existed to increase inter-rater reliability on the QA assessments, it is not possible to entirely eliminate subjectivity from scoring. Ideally, data collection should have taken place in peak VMMC periods in order to evaluate efficiency elements under high volume conditions; however, due to delays in obtaining approvals and logistical problems of scheduling, these data reflect a mix of low, medium and high volume conditions. In addition, since this study did not include a control group, the decrease in quality may not be attributable to the rapid scale-up of the program alone since other factors could have influenced program quality. Lastly, the expanded site comparisons were affected by collinearity, as data from the same 15 sites in 2011 and 2012 were used in these analyses.

## References

[1] UNAIDS (2010) Country Overview: South Africa. Geneva: UNAIDS.

[2] Auvert B, Taljaard D, Lagarde E, Sobngwi-Tambekou J, Sitta R, et al (2005) Randomized, controlled intervention trial of male circumcision for reduction of HIV infection risk: the ANRS 1265 Trial. PLoS Med 2: e298. 16231970 10.1371/journal.pmed.0020298 PMC1262556

[3] Bailey RC, Moses S, Parker CB, Agot K, Maclean I, et al (2007) Male circumcision for HIV prevention in young men in Kisumu, Kenya: a randomised controlled trial. Lancet 369: 643–656. 17321310 10.1016/S0140-6736(07)60312-2

[4] Gray RH, Kigozi G, Serwadda D, Makumbi F, Watya S, et al (2007) Male circumcision for HIV prevention in men in Rakai, Uganda: a randomised trial. Lancet 369: 657–666. 17321311 10.1016/S0140-6736(07)60313-4

[5] World Health Organization, UNAIDS (2007) New data on male circumcision and HIV prevention: policy and programme implications: WHO/UNAIDS Technical Consultation Male Circumcision and HIV Prevention: Research Implications for Policy and Programming, Montreux, 6–8 March 2007: Conclusions and recommendations. Geneva: World Health Organization. 9789241595988. 15 p.

[6] Johnson S, Kincaid DL, Laurence S, Chikwava F, Delate R, et al.. (2010) Second National HIV Communication Survey 2009. Pretoria: JHHESA.

[7] Statistics South Africa (2012) Mid-year population estimates, 2011. Pretoria: Statistics South Africa.

[8] South Africa Department of Health (2011) Implementation plan for the scale up of medical male circumcision (MMC) programme in South Africa: 2011–2016. Unpublished report.

[9] Njeuhmeli E, Forsythe S, Reed J, Opuni M, Bollinger L, et al (2011) Voluntary medical male circumcision: modeling the impact and cost of expanding male circumcision for HIV prevention in eastern and southern Africa. PLoS Med 8: e1001132. 22140367 10.1371/journal.pmed.1001132 PMC3226464

[10] Auvert B, Taljaard D, Sitta R, Rech D, Lissouba P, et al.. (2012) Decrease of HIV prevalence over time among the male population of Orange Farm, South Africa, following a circumcision roll-out (ANRS-12126). Washington, D.C.

[11] World Health Organization (2010) Considerations for implementing models for optimizing the volume and efficiency of male circumcision services. Geneva: World Health Organization.

[12] World Health Organization (2008) Male circumcision quality assurance: a guide to enhancing the safety and quality of services. Geneva: World Health Organization. 9789241597319. 68 p.

[13] Oliver K, Young M, Oliphant N, Diaz T, Kim J (2012) Review of systematic challenges to the scale-up of integrated community case management: Emerging lessons & recommendations from the catalytic initiative (CI/IHSS). New York: United Nations Children's Fund (UNICEF).

[14] World Health Organization (2006) Quality of care: a process for making strategic choices in health systems. Geneva: World Health Organization. 9241563249. 38 p.

[15] World Health Organization (2012) Progress in scaling up voluntary medical male circumcision for HIV prevention in East and Southern Africa: January–December 2011. Geneva: World Health Organization.

[16] Bertrand JT, Rech D, Dickens O, Frade S, Loolpapit M, et al (2013) SYMMACS: Systematic monitoring of the male circumcision scale-up in Eastern and Southern Africa, full report of results from Kenya, South Africa, Tanzania, and Zimbabwe. PLoS Med In press.

[17] World Health Organization (2009) Male circumcision services: quality assessment toolkit. Geneva: World Health Organization. 9789241597517. 127 p.

[18] Bertrand JT, Rech D, Dickens O, Frade S, Loolpapit M, et al.. (2012) Systematic monitoring of male circumcision scale-up in Eastern and Southern Africa: Interim report of results from Kenya, South Africa, Tanzania, and Zimbabwe. Baltimore: Johns Hopkins School of Public Health.

[19] Horizons Program, YRG CARE, International HIV/AIDS Alliance (2004) Expanding Care and Support in South India: Scaling Up YRG CARE's Patient-centered Approach. Washington, DC: Population Council.

[20] Fajans P, Thom NT, Whittaker M, Satia J, Mai TTP, et al.. (2007) Strategic choices in scaling up: introducing injectable contraception and improving quality of care in Viet Nam. Scaling up Health Service Delivery: From Pilot Innovations to Policies and Programmes Geneva: WHO: 31–52.

[21] Bertrand JT, Rech D, Dickens O, Frade S, Loolpapit M, et al (2013) Systematic monitoring of the voluntary medical male circumcision scale-up: adoption of efficiency elements in Kenya, South Africa, Tanzania, and Zimbabwe. PLoS Med In press. 10.1371/journal.pone.0082518 PMC4011576 24801374

[22] Rech D, Bertrand JT, Thomas NJ, Farrell-Ross M, Reed J, et al (2013) Surgical efficiencies and quality in the performance of voluntary medical male circumcision (VMMC) procedures in Kenya, South Africa, Tanzania, and Zimbabwe. PLoS Med In press. 10.1371/journal.pone.0084271 PMC4011873 24802412

